# An AI-assisted tool for automated growth monitoring in pediatric achondroplasia

**DOI:** 10.1007/s00431-025-06321-3

**Published:** 2025-07-18

**Authors:** Eyal Cohen-Sela, Yael Lebenthal, Avivit Brener, Ravit Regev, Lars Hagenäs

**Affiliations:** 1 The Institute of Pediatric Endocrinology, Diabetes and Metabolism, Dana-Dwek Children’s Hospital, Tel Aviv Sourasky Medical Center, 64239-06 Tel Aviv, Israel; 2 The School of Medicine, Gray Faculty of Medical & Health Sciences, Tel Aviv University, Tel Aviv, Israel; 3 Department of Women’s and Children’s Health, Karolinska Institutet, Stockholm, Sweden

**Keywords:** Achondroplasia, Anthropometric measurements, Artificial intelligence (AI), Electronic growth charts, Growth assessment, *Z*-scores

## Abstract

Growth assessment in achondroplasia requires disorder-specific growth charts incorporating sex- and age-specific values. Manual calculations are tedious and subject to error. We present an artificial intelligence (AI)-assisted tool that automates *z*-score calculations for pediatric patients with achondroplasia. The tool integrates European Lambda-Mu-Sigma (LMS) growth reference data for 9 anthropometric parameters: height, weight, body mass index, head circumference, sitting height, leg length, arm span, relative sitting height, and foot length. It inputs anthropometric measurements and transforms them into sex- and age-specific *z*-scores and percentiles in real time. Ten pediatric endocrinologists independently calculated anthropometric *z*-scores for 3 patients with achondroplasia using both the manual growth charts and the automated tool. Time-to-completion and accuracy were recorded and compared. The mean time required by the AI-assisted tool to calculate *z*-scores for all 9 parameters was significantly shorter than that required by manual calculation (23.4 ± 5.8 vs. 10.1 ± 2.8 min, *p* < 0.001). The tool demonstrated 100% agreement with manual LMS-based calculations and eliminated human errors to which manual calculations are subject, with significantly higher median absolute *z*-score deviation compared to the smart tool (0.17 [0.07–0.30] vs. 0 [0–0.01], *p* < 0.001).

*Conclusion*:This AI-assisted tool provides a user-friendly, accessible, and highly accurate method for automated growth assessment in pediatric achondroplasia. It facilitates efficient clinical and research applications, with potential for future integration into electronic health records and web-based platforms.

**Table:** 

**What is Known:** •*Growth monitoring in achondroplasia requires syndrome-specific Lambda-Mu-Sigma based charts.* •*Manual z-score calculations are time-consuming and subject to error.*	
**What is New:** •*We present an AI-assisted Excel tool that automates z-scores and percentile calculations for 9 anthropometric parameters.* •*Performance and inter-user reliability testing by 10 pediatric endocrinologists showed significantly improved speed and accuracy over manual methods.*

**What is Known:**

•*Growth monitoring in achondroplasia requires syndrome-specific Lambda-Mu-Sigma based charts.*

•*Manual z-score calculations are time-consuming and subject to error.*

**What is New:**

•*We present an AI-assisted Excel tool that automates z-scores and percentile calculations for 9 anthropometric parameters.*

•*Performance and inter-user reliability testing by 10 pediatric endocrinologists showed significantly improved speed and accuracy over manual methods.*

## Introduction

Achondroplasia is the most common cause of disproportionate short stature [1]. Accurate growth monitoring in this population requires the use of syndrome-specific growth charts, since standard references do not reflect their unique growth trajectories [2]. These charts enable clinicians to assess height, weight, head circumference, and body proportions, all of which are parameters critical for identifying deviations from expected growth and for guiding timely interventions.

Until recently, therapeutic options for increasing height in children with achondroplasia were limited to growth hormone therapy and surgical limb lengthening, the latter involving an invasive procedure associated with considerable risks and complications. In 2021, the United States Food and Drug Administration approved vosoritide, a C-type natriuretic peptide analog, as the first pharmacological treatment specifically targeting the underlying growth impairment in achondroplasia. This approval has led to an increase in pediatric endocrinology consultations, with more families seeking vosoritide treatment for their children [3, 4].

The Lambda-Mu-Sigma (LMS) method is widely used to generate growth curves for anthropometric parameters [5]. In the setting of achondroplasia, LMS-based charts have been developed for specific parameters, such as sitting height, leg length, and other proportional measurements, which are especially relevant for monitoring skeletal growth, and for identifying deviations from expected growth patterns and potential complications [6–10].

Despite the availability of well-validated, European LMS-based growth charts for achondroplasia, their use in clinical practice is often limited by the complexity and time required for manual *z*-score calculations [11]. This process is not only inefficient but also subject to human error. We developed a novel artificial intelligence (AI)-assisted tool that automates *z*-score and percentile calculations for pediatric patients with achondroplasia. The tool offers a rapid, reliable, and user-friendly solution for assessing growth and for improving the implementation of syndrome-specific monitoring in both clinical and research settings.

## Methods

### Tool development

We developed an Excel-based tool to automate LMS-based *z*-score calculations for pediatric patients with achondroplasia. The tool integrates sex- and age-specific European growth charts for multiple anthropometric parameters [9, 10]. Height, weight, body mass index (BMI), and head circumference were included from birth to 20 years, with 6-month intervals up to age 4 years and 1-year intervals thereafter. Sitting height, leg length, arm span, and relative sitting height were assessed from ages 2–20 years, while foot length was evaluated from ages 3–20 years, all with 1-year intervals.

**Fig. 1:**
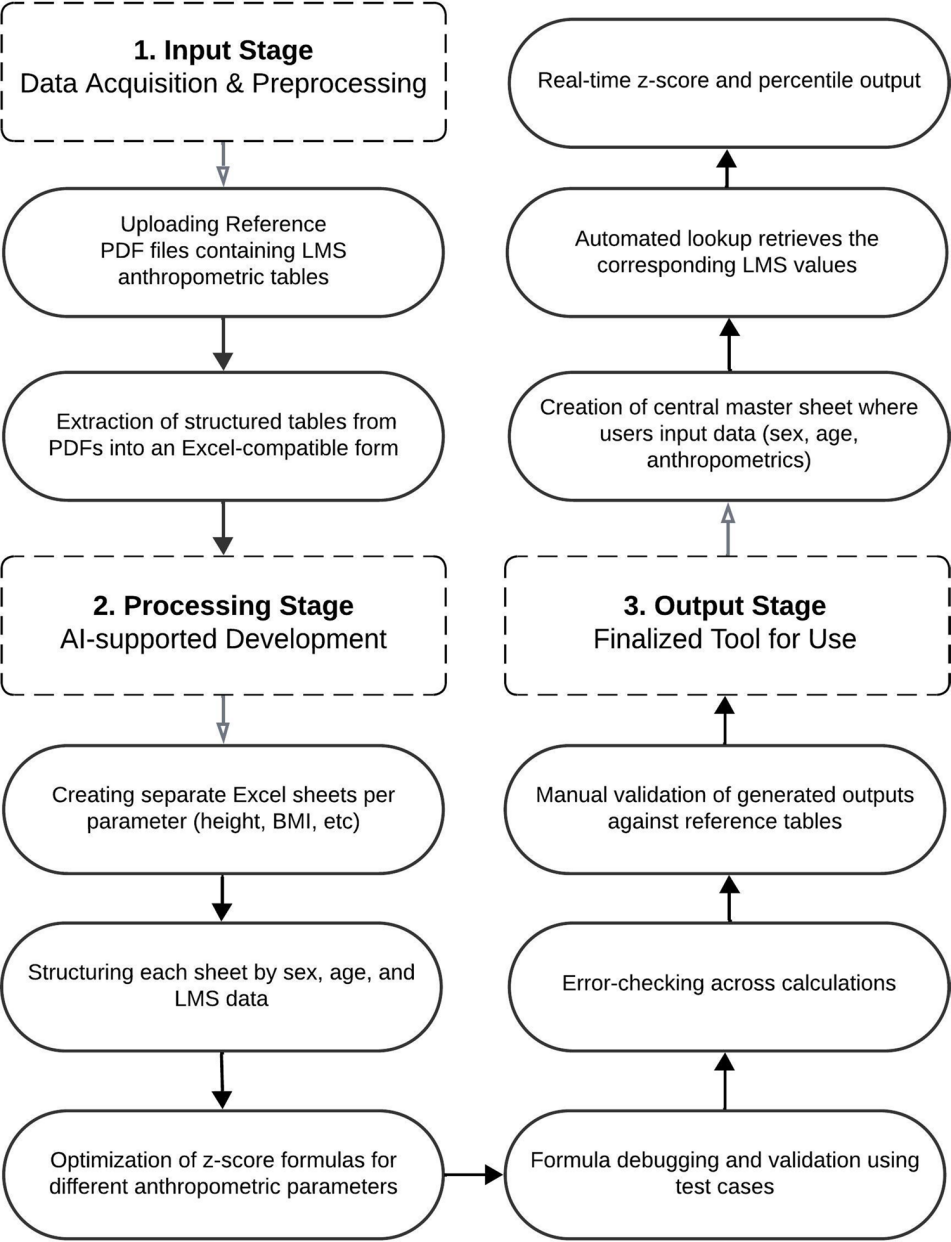
AI-assisted workflow for automating anthropometric *z*-score calculations using LMS data

The AI-assisted approach utilized large language models (specifically, OpenAI’s GPT 4o-based architecture) to enhance tool development [12]. The AI system assisted with extracting data from reference tables and coding complex Excel formulas. AI optimization was particularly useful for age-specific interpolation. The clinical team supervised the process throughout the AI-assisted development of the tool (Fig. 1).

The tool dynamically extracts the appropriate LMS values for each parameter. For parameters where *L* = 1 (height, head circumference, sitting height, leg length, arm span, relative sitting height, and foot length), the *z*-scores were calculated using the standard formula:

$$Z=\left(X-M\right)/S$$

For non-normally distributed parameters, including weight and BMI, the full LMS box-cox transformation was applied:

$$Z=\left(\left({\textstyle\frac XM}\right)^L-1\right)/L\times S$$

where *X* represents the patient’s individual measurement. An interpolation function was implemented for ages not directly listed in the reference tables in order to ensure accurate calculations across all age groups.

### Validation and reliability assessments

Manual LMS-based calculations were conducted for multiple test cases in order to verify the tool’s accuracy. A validation dataset comprising 30 patients randomly sampled across sexes and age groups was used to compare manual and automated *z*-score outputs. Interpolation points between age intervals were included to assess the degree of precision across the pediatric age range. Extreme values were also tested to evaluate the tool’s robustness at percentile boundaries.

To assess inter-user reliability and performance, 10 pediatric endocrinologists from our institution were asked to independently calculate *z*-scores and percentiles for 3 patients. Each physician performed 54 calculations (9 anthropometric parameters per patient carried out manually and by using the AI-assisted tool). The deviation from the reference gold standard (defined as the AI-assisted tool used by its developer) was recorded for each calculation. The median absolute deviation and mean total calculation time were compared between the manual and automated methods. Group-level and individual-level comparisons were performed using the Mann–Whitney-Wilcoxon test.

## Results

The tool demonstrated complete agreement with the LMS-based calculations across all anthropometric parameters, including at interpolation points and percentile extremes. The AI-assisted tool significantly reduced the time required to compute *z*-scores and percentiles for all 9 parameters compared to manual methods (10.1 ± 2.8 vs. 23.4 ± 5.8 min, respectively, *p* < 0.001). The median absolute *z*-score deviation across all raters and cases was significantly lower using the AI-assisted tool in the inter-rater reliability assessment (0 [0–0.01]) compared to manual calculations (0.17 [0.07–0.30], *p* < 0.001). These findings demonstrate consistent improvements in accuracy and precision across all evaluators and test cases (Table 1).

**Table 1:** Median z-score deviations for anthropometric measurements across 3 cases calculated manually and using the smart AI-assisted tool

	Case 1			Case 2			Case 3			Total		
Rater	Manual	Smart		Manual	Smart		Manual	Smart		Manual	Smart	*p*
1	0.30 [0.13–0.40]	0 [0–0]		0.17 [0.12–0.36]	0 [0–0.01]		0.10 [0.05–0.11]	0 [0–0.01]		0.13 [0.09–0.33]	0 [0–0.01]	**< 0.001**
2	0.20 [0.07–0.30]	0 [0–0]		0.08 [0.06–0.10]	0.01 [0–0.01]		0.15 [0.10–0.21]	0 [0–0.01]		0.10 [0.06–0.22]	0 [0–0.01]	**< 0.001**
3	0.40 [0.13–0.76]	0 [0–0]		0.26 [0.17–0.56]	0 [0–0]		0.20 [0.10–0.42]	0 [0–0]		0.21 [0.16–0.54]	0 [0–0]	**< 0.001**
4	0.40 [0.30–0.60]	0.04 [0.02–0.15]		0.28 [0.11–0.17]	0.02 [0–0.05]		0.12 [0.09–0.31]	0.02 [0–0.04]		0.30 [0.11–0.70]	0.03 [0–0.05]	**< 0.001**
5	0.20 [0.13–0.30]	0 [0–0]		0.07 [0.06–0.26]	0 [0–0]		0.10 [0.05–0.21]	0 [0–0]		0.13 [0.07–0.27]	0 [0–0]	**< 0.001**
6	0.24 [0.13–0.3]	0.02 [0–0.04]		0.17 [0.06–0.26]	0.03 [0.01–0.03]		0.11 [0.05–0.16]	0.01 [0–0.02]		0.17 [0.08–0.25]	0.02 [0–0.03]	**< 0.001**
7	0.30 [0.30–0.50]	0 [0–0.01]		0.17 [0.06–0.30]	0 [0–0.02]		0.10 [0.05–0.21]	0 [0–0.01]		0.22 [0.10–0.31]	0 [0–0.01]	**< 0.001**
8	0.30 [0.20–0.38]	0 [0–0]		0.07 [0.04–0.11]	0.01 [0–0.03]		0.05 [0.01–0.11]	0.01 [0.01–0.02]		0.11 [0.04–0.24]	0 [0–0.02]	**< 0.001**
9	0.32 [0.18–0.40]	0 [0–0.01]		0.16 [0.10–0.21]	0.01 [0–0.03]		0.05 [0–0.07]	0.01 [0–0.02]		0.16 [0.06–0.22]	0 [0–0.03]	**< 0.001**
10	0.22 [0.20–0.30]	0 [0–0]		0.17 [0.07–0.21]	0 [0–0.01]		0.1 [0.05–0.20]	0 [0–0]		0.20 [0.10–0.23]	0 [0–0]	**< 0.001**

	Case 1			Case 2			Case 3			**Total**		
All	Manual	Smart	*p*	Manual	Smart	*p*	Manual	Smart	*p*	Manual	Smart	*p*
	0.30 [0.15–0.40]	0 [0–0.01]	**< 0.001**	0.17 [0.07–0.30]	0 [0–0.03]	**< 0.001**	0.10 [0.05–0.21]	0 [0–0.01]	**< 0.001**	0.17 [0.07–0.30]	0 [0–0.01]	**<0.001**

The data are expressed as median [interquartile range bounds]. Values reflect the median absolute z-score deviation from the gold-standard reference, defined as the output of the AI-assisted tool when used by the tool’s developer. The column labeled ‘total’ summarizes deviations across all 3 cases per evaluator. The row labeled “all” summarizes deviations across all evaluators. **Bold** indicates statistical significance at a *p* ≤ 0.05 level (Mann-Whitney-Wilcoxon test)

## Discussion

We introduce an AI-assisted tool designed to automate *z*-score calculations for pediatric patients with achondroplasia. Our application of the tool demonstrates its capability of providing a more efficient, accurate, and user-friendly alternative to traditional manual methods. By automating LMS-based calculations, the tool reduces clinician workloads and minimizes potential errors, thereby facilitating real-time decision-making essential for monitoring growth velocity, assessing treatment responses and identifying the need for early interventions.

Many pediatric endocrinology clinics continue to use either locally relevant or U.S.-based growth references for monitoring achondroplasia. This AI-assisted tool offers accurate assessments by integrating European-specific growth charts. Unlike general growth calculators, such as PediTools and World Health Organization Anthro, which lack achondroplasia-specific LMS parameters, this tool directly incorporates European achondroplasia growth standards. It operates offline, thereby eliminating the need for internet connectivity, and is customizable for both research and clinical applications. Unlike some existing electronic health record (EHR) systems, this tool includes growth tracking modules specialized for parameters most relevant to achondroplasia. Since it is currently limited to European reference data and may not be fully generalizable to non-European populations, future enhancements integrating additional datasets from other populations are warranted to broaden its applicability [1, 6, 7]. Additionally, the tool does not yet support direct integration with EHR systems. Privacy considerations for this offline tool are minimal, as no personal health information is transmitted or stored externally. Nonetheless, adherence to best practices in data protection remains essential, particularly if future versions incorporate integration with electronic health records or cloud-based features [13].

In conclusion, this AI-assisted Excel tool automates *z*-score and percentile calculations for pediatric achondroplasia, ensuring accuracy, efficiency, and accessibility. It eliminates manual errors and provides real-time assessments, making it a potentially invaluable resource for clinicians and researchers.

## Data Availability

No datasets were generated or analysed during the current study.

## Abbreviations

AI: Artificial intelligence
BMI: Body mass index
EHR: Electronic health record
LMS: Lambda-Mu-Sigma
